# A modified indirect method for computing inbreeding coefficients

**DOI:** 10.1186/s12711-026-01035-y

**Published:** 2026-02-22

**Authors:** Che Hsuan Huang, Seijiro Hirama, Toshimi Baba, Junpei Kawakami, Takeshi Yamazaki, Koichi Hagiya

**Affiliations:** 1 Division of Dairy Production Research, Hokkaido Agricultural Research Center, NARO, Sapporo, 062-8555 Japan; 2 Department of Life and Food Science, Obihiro University of Agriculture and Veterinary Medicine, Obihiro, 080-8555 Japan; 3 Holstein Cattle Association of Japan, Hokkaido Branch, Sapporo, 001-8555 Japan

## Abstract

The performance of existing methods for computing inbreeding coefficients—including tabular-based methods, Cholesky decomposition-based methods, and indirect methods—has been limited by either memory usage, pedigree depth, or average half-sib family size. In this note, we present a modified indirect method that efficiently identifies, computes, and stores the necessary elements for computing inbreeding coefficients. The algorithm was tested on pedigrees of 8.6 million Japanese Holsteins born from 1901 to 2023, with an average longest ancestral path number of 17 and 42,120 sires with progenies. Compared to a previous indirect method, the modifications reduced computation time from 103.4 to 7.2 s. When parallelized using 32 threads, the computation completed in 1.1 s. Simulation results suggested that the proposed method is less affected by pedigree depth or family size, making it practical for most livestock populations reproduced via artificial insemination.

**Supplementary Information:**

The online version contains supplementary material available at 10.1186/s12711-026-01035-y.

## Background

Despite the growing use of genomic measures of inbreeding, traditional pedigree-based inbreeding coefficients (F) defined by Wright [1] remain essential in genetic analyses of livestock. These coefficients are used to construct the inverse of the numerator relationship matrix (${\mathbf{A}}^{-1}$) in the mixed model equations [2] and to derive the accuracy of estimated breeding values [3], even in so-called single-step genomic evaluations [4]. They are also required for optimizing mating plans [5], whether to preserve genetic diversity [6, 7] or to reduce the risk of deleterious effects from homozygosity of recessive alleles [8].

In well-managed livestock populations, pedigrees accumulate over generations, making the computation of F increasingly time-consuming. When pedigrees are incomplete or ancestral allele frequencies are unknown, F needs to be computed repeatedly across iterations [9, 10], creating potential computational bottlenecks [5]. Several algorithms have been proposed to compute F in large populations [5, 9, 11–15], which can be broadly categorized into three types.

The algorithms proposed by Tier [11], among others [5, 9], are efficient implementations of the *tabular method* [16]. The algorithms use recursive functions to trace pedigrees from descendants to ancestors, computing only the necessary elements of **A** and storing them for repeated use. As the number of required elements grows quadratically with the population size, additional effort is required to determine which elements to retain in memory to meet physical memory constraints [5].

The second type of algorithm is based on the *Cholesky decomposition* of $\mathbf{A}=\mathbf{L}\mathbf{D}{\mathbf{L}}^{\mathbf{^{\prime}}}$ [2, 12, 15, 17]. When the pedigrees are ordered such that parents precede offspring, $\mathbf{L}$ is a lower triangular matrix representing the fraction of genes that each animal inherits from its ancestors, and $\mathbf{D}$ is a diagonal matrix containing the within-family additive genetic variances of animals. The F of animal *i* can thus be computed using only the i-th row of $\mathbf{L}$ and the diagonal elements of $\mathbf{D}$, i.e., ${{\mathrm{F}}_{\mathrm{i}}=\mathrm{A}}_{\mathrm{i,i}}-1=(\sum_{\mathrm{j}=1}^{\mathrm{i}}{\mathrm{L}}_{i,j}^{2}{\mathrm{D}}_{j,j})-1$. In the algorithm by Meuwissen and Luo [12], the elements of $\mathbf{L}$ are stored only temporarily, such that most of the data remain in the CPU cache, which are fast to access. However, the computation cost of the algorithm grows exponentially with the number of generations, making it less efficient with deep pedigrees.    

The third type of algorithm is the *indirect method* proposed by Colleau [14], later refined by Sargolzaei et al. [13]. The indirect method calculates the F of progenies of a sire indirectly by solving the linear system ${\mathbf{A}}^{-1}\mathbf{y}={(\mathbf{L}}^{-1})\mathbf{^{\prime}}{\mathbf{D}}^{-1}{\mathbf{L}}^{-1}\mathbf{y}=\mathbf{x}$, where $\mathbf{x}$ is a sparse vector with a single 1 in the position corresponding to the sire. Accordingly, the solution $\mathbf{y}=\mathbf{A}\mathbf{x}$ gives the column of $\mathbf{A}$ for the sire, from which the F of his offspring can be inferred. This algorithm takes advantage of the sparsity of ${\mathbf{L}}^{-1}$ and the presence of huge half-sib families common in livestock populations, but such efficiency diminishes if the population consists of many small families.

Based on simulation results, Sargolzaei et al. [13] reported that the indirect method was the fastest among the available algorithms. However, in our preliminary analysis of a real Holstein pedigree, the algorithm was slower than another algorithm based on the Cholesky decomposition of $\mathbf{A}$ by Sargolzaei and Iwaisaki [15]. A plausible explanation is that the real pedigrees may contain many sires and animals but span relatively fewer generations, significantly diminishing the advantages of Sargolzaei et al.’s indirect method [13].

In this study, we modified the indirect method to compute F quickly, even for large populations consisting of many small families. The algorithm incorporates the metafounder approach to model unknown parents [18, 19], and strategies for parallelization were also implemented. To evaluate its performance relative to previous algorithms, we used a real Holstein pedigree along with several simulated pedigrees.

## Materials and methods

### Algorithm

#### Sorting the pedigree

We started the algorithm by assigning unknown parents to metafounders [18] and sorting the pedigree in ascending order of the longest ancestral path (LAP) number [15], defined as:

1 $${\mathrm{LAP}}_{{\mathrm{i}}} = \left\{ {\begin{array}{*{20}l} {0, } \hfill & \text{if i is a metafounder,} \hfill \\ {\max \left( {{\mathrm{LAP}}_{{{\mathrm{s}}_{{\mathrm{i}}} }} ,{\mathrm{LAP}}_{{{\mathrm{d}}_{{\mathrm{i}}} }} } \right) + 1,} \hfill & \text{if i is a real animal, } \hfill \\ \end{array} } \right. $$

where ${s}_{i}$ and ${d}_{i}$ represent the sire and dam of animal *i*, respectively. This ensured that metafounders appeared at the beginning of the pedigree and that all parents precede their progeny.

#### Modifying the indirect method

To get $\mathbf{y}=\mathbf{A}\mathbf{x}$, the linear system ${{\mathbf{A}}^{-1}\mathbf{y}=(\mathbf{L}}^{-1}){^{\prime}}{\mathbf{D}}^{-1}{\mathbf{L}}^{-1}\mathbf{y}=\mathbf{x}$ can be solved in 2 steps [14]:

1. Backward substitution to solve , 
2. Forward substitution to solve ${\mathbf{L}}^{-1}\mathbf{y}=\mathbf{D}\mathbf{z}$,

where ${\mathbf{L}}^{-1}$ is a sparse lower triangular matrix with 1 on the diagonal and at most two values of -0.5 per row to link each animal to its parents, ${\mathbf{D}}^{-1}$ is the inverse of $\mathbf{D}$, and $\mathbf{z}={\mathbf{D}}^{-1}{\mathbf{L}}^{-1}\mathbf{y}$.

Sargolzaei et al. [13] implemented the backward substitution step by computing nonzero elements in $\mathbf{z}$ from the position of the target sire, say $s$, to the beginning of the pedigree. This results in a computational cost that increases linearly with the pedigree size. To improve efficiency, they prepared a reduced pedigree containing only sires to be evaluated, the sires’ mates, and all their ancestors. However, in populations with many genetic origins, the vector ${\mathbf{z}}^{\mathbf{r}}$ (where the superscript r indicates that the array contains values only for animals in the reduced pedigree) can still be long and sparse, especially when family lines are short or inbred. Alternatively, because $\mathbf{x}$ is a unit vector with 1 at the position corresponding to the sire $s$, the backward substitution can be simplified as:

2 $$\mathbf{z}={\mathbf{L}}{{^{\prime}}}\mathbf{x}={\mathbf{L}^{\prime}_{\mathbf{s}, :}},$$

where ${\mathbf{L}}_{\mathbf{s}, :}$ denotes the *s*-th row of $\mathbf{L}$. This allows us to bypass the backward substitution step by directly computing ${\mathbf{L}}_{\mathbf{s}, :}$, using either Meuwissen and Luo’s [12] or Sargolzaei and Iwaisaki’s [15] method. We adopted the latter, as it evaluates ancestors in descending order of LAP numbers rather than identification numbers, minimizing the cost of sorting the ancestor list for computing ${\mathbf{L}}_{\mathbf{s}, :}$. Once an element of ${\mathbf{L}}_{\mathbf{s}, :}$ had been computed, it was multiplied by the corresponding diagonal element of $\mathbf{D}$.

For the forward substitution step, Sargolzaei et al. [13] computed the elements of ${\mathbf{y}}^{\mathbf{r}}$, i.e., the *s*-th column of the reduced matrix ${\mathbf{A}}^{\mathbf{r}}$, by the following procedure:

3 $$ {\text{For i}} = 1{\text{ to mip}}_{{\mathrm{s}}} ,{\text{ y}}_{{\mathrm{i}}}^{{\mathrm{r}}} = \frac{1}{2}\left( {{\mathrm{y}}_{{{\mathrm{s}}_{{\mathrm{i}}} }}^{{\mathrm{r}}} + {\mathrm{y}}_{{{\mathrm{d}}_{{\mathrm{i}}} }}^{{\mathrm{r}}} } \right) + {\mathrm{D}}_{{{\mathrm{i}},{\mathrm{i}}}}^{{\mathrm{r}}} {\mathrm{z}}_{{\mathrm{i}}}^{{\mathrm{r}}} { }, $$

where ${mip}_{s}$ refers to the mate of sire *s* with the largest identification number in the reduced pedigree, and ${s}_{i}$​ and ${d}_{i}$​ denote the sire and dam of *i*, respectively. These values were then halved to derive F for the progeny between the sire *s* and all his mates.

However, similar to the backward substitution step, the procedure in Eq. (3) results in a computational cost that increases linearly with the size of the reduced pedigree, and for sires with few mates, many of the computed values are unused. To address this, we implemented a recursive function that computes only the necessary elements of $\mathbf{y}$ corresponding to the mates of sire s as well as their ancestors. Accordingly, redundant calculations were avoided by holding a logical vector, say $\mathbf{f}\mathbf{l}\mathbf{a}\mathbf{g}$, to flag whether each element of $\mathbf{y}$ had already been computed. $\text{For }i\in {\mathbf{M}\mathbf{A}\mathbf{T}\mathbf{E}}_{\mathbf{s}}$:

4 $$\begin{aligned} & {\mathrm{For}}\;{\mathrm{i}} \in {\mathbf{MATE}}_{{\mathbf{s}}} , \\ & \left\{ {\begin{array}{*{20}l} {if\;{\mathrm{flag}}_{{\mathrm{i}}} = FALSE\;then\;{\mathrm{y}}_{{\mathrm{i}}}^{{}} = \frac{1}{2}\left( {{\mathrm{y}}_{{{\mathrm{s}}_{{\mathrm{i}}} }}^{{}} + {\mathrm{y}}_{{{\mathrm{d}}_{{\mathrm{i}}} }}^{{}} } \right) + {\mathrm{D}}_{{{\mathrm{i}},{\mathrm{i}}}}^{{}} {\mathrm{z}}_{{\mathrm{i}}}^{{}} ,{\mathrm{flag}}_{{\mathrm{i}}} = TRUE} \hfill \\ {if\;{\mathrm{flag}}_{{\mathrm{i}}} = TRUE\;then\;{\mathrm{y}}_{{\mathrm{i}}}^{{}} = {\mathrm{y}}_{{\mathrm{i}}}^{{}} } \hfill \\ \end{array} } \right. \\ \end{aligned} $$

where ${\mathbf{M}\mathbf{A}\mathbf{T}\mathbf{E}}_{\mathbf{s}}$ denotes the list of the mates of sire *s*, and ${y}_{{s}_{i}}$ and ${y}_{{d}_{i}}$ were computed recursively by calling the same function with *i* replaced by ${s}_{i}$​ or ${d}_{i}$​, respectively. A similar recursive function was also used to initialize the vectors $\mathbf{y}$ and $\mathbf{f}\mathbf{l}\mathbf{a}\mathbf{g}$. This approach eliminates the effect of pedigree size on computation performance, removing the need to create a reduced pedigree or to identify ${mip}_{s}$.

Interestingly, our approach reveals that the indirect method for computing F is also based on the Cholesky decomposition of $\mathbf{A}$. It is known [12, 20] that:

5 $${\mathrm{F}}_{\mathrm{j}}=\frac{1}{2}{\mathrm{A}}_{{\mathrm{d}}_{\mathrm{j}},\mathrm{s}}=\frac{1}{2}{\mathbf{L}}_{{\mathbf{d}}_{\mathbf{j}},:}\mathbf{D}{\mathbf{L}}_{\mathbf{s},:}^{\prime}=\frac{1}{2}\sum_{\mathrm{k}\in \mathbf{A}\mathbf{N}{\mathbf{C}}_{\mathbf{s}}\cap {\mathbf{A}\mathbf{N}\mathbf{C}}_{{\mathbf{d}}_{\mathbf{j}}}}{\mathrm{L}}_{{\mathrm{d}}_{\mathrm{j}},\mathrm{k}}{\mathrm{D}}_{\mathrm{k},\mathrm{k}}{\mathrm{L}}_{\mathrm{s},\mathrm{k}},$$

where ${d}_{j}$ is the dam of *j* (i.e., a mate of *s*), and $\mathbf{A}\mathbf{N}{\mathbf{C}}_{\mathbf{s}}$ and $\mathbf{A}\mathbf{N}{\mathbf{C}}_{{\mathbf{d}}_{\mathbf{j}}}$ represent the lists of the ancestors of $s$and of ${d}_{j}$, respectively. The animal itself is also included in the list.

Since $\mathbf{D}\mathbf{z}\equiv \mathbf{D}{\mathbf{L}}_{\mathbf{s},:}^{\prime}$, the initial values of vector $\mathbf{y}$ contain ${\mathrm{D}}_{\mathrm{m},\mathrm{m}}{\mathrm{L}}_{\mathrm{s},\mathrm{m}}$ for $m\in \mathbf{A}\mathbf{N}{\mathbf{C}}_{\mathbf{s}}$. The recursive function in Eq. (4) thus recursively traces back the pedigree of ${d}_{j}$ to search for co-ancestry between $s$ and ${d}_{j}$. For any $n\in \mathbf{ANC}_{\mathbf{d}_{\mathbf{j}}}$:

6 $$\begin{aligned} {\mathrm{y}}_{{\mathrm{n}}} & = \frac{1}{2}\left( {{\mathrm{y}}_{{{\mathrm{s}}_{{\mathrm{n}}} }} + {\mathrm{y}}_{{{\mathrm{d}}_{{\mathrm{n}}} }} } \right) + {\mathrm{D}}_{{{\mathrm{n}},{\mathrm{n}}}} {\mathrm{L}}_{{{\mathrm{s}},{\mathrm{n}}}} \\ & = \frac{1}{2}\left( {{\mathbf{L}}_{{{\mathbf{s}}_{{\mathbf{n}}} ,:}} {\mathbf{DL}}_{{{\mathbf{s}},:}}^{\prime} + {\mathbf{L}}_{{{\mathbf{d}}_{{\mathbf{n}}} ,:}} {\mathbf{DL}}_{{{\mathbf{s}},:}}^{\prime} } \right) + {\mathrm{D}}_{{{\mathrm{n}},{\mathrm{n}}}} {\mathrm{L}}_{{{\mathrm{s}},{\mathrm{n}}}} \\ & = {\mathbf{L}}_{{{\mathbf{n}},:}} {\mathbf{DL}}_{{{\mathbf{s}},:}} = {\mathrm{A}}_{{{\mathrm{n}},{\mathrm{s}}}} , \\ \end{aligned} $$

where ${d}_{n}$ and ${s}_{n}$ are the dam and the sire of $n$, and ${\mathrm{D}}_{\mathrm{n},\mathrm{n}}{\mathrm{L}}_{\mathrm{s},\mathrm{n}}$ would be zero if $n\notin \mathbf{A}\mathbf{N}{\mathbf{C}}_{\mathbf{s}}$.        

It is instructive to compare the proposed algorithm with that of Quaas, as described in Appendix B2 of Mrode (2014) [20]. Quaas' algorithm uses the same expression as Eq. (5) to compute ${\mathrm{F}}_{\mathrm{j}}$, but focuses on identifying common ancestors *k* of sire $s$ and dam ${d}_{j}$ (i.e.,$k\in \mathbf{A}\mathbf{N}{\mathbf{C}}_{\mathbf{s}}\cap {\mathbf{A}\mathbf{N}\mathbf{C}}_{{\mathbf{d}}_{\mathbf{j}}}$). Importantly, in that approach, the *s*-th row of $\mathbf{L}$ (i.e., ${\mathbf{L}}_{\mathbf{s},:}$) is not stored, and the $\mathrm{F}$ for each progeny *j* is computed independently. As a consequence, the method cannot efficiently exploit the presence of large half-sib families, which are common in livestock populations.

In contrast, the proposed method explicitly stores ${\mathbf{L}}_{\mathbf{s},:}$ and recursively traces the pedigrees of all corresponding dams ${d}_{j}$ for the progenies of sire *s* (i.e., ${\mathbf{M}\mathbf{A}\mathbf{T}\mathbf{E}}_{\mathbf{s}}$). When only a single dam is present, tracing its pedigree requires approximately the same time as computing ${\mathbf{L}}_{\mathbf{s},:}$. However, when multiple dams are present, the recursive function in Eq. (4) stores the computed coefficients at position *k* of the vector **y** for all $k\in {\bigcup }_{\mathbf{j}}\mathbf{ANC}_{\mathbf{d}_{\mathbf{j}}}$ (i.e., any ancestor of any ${d}_{j}$), and redundant computations are avoided when coancestry exists among the dams.

#### Parallelization

Animals with identical LAP numbers share no parent–offspring relationships, and thus their F can be computed independently. As the pedigree was sorted in ascending order of LAP numbers, parallelization can be implemented by including an inner parallel loop within each LAP block.

### Data for the evaluation of performance

#### Pedigree of Japanese Holsteins

The pedigree data of Japanese Holsteins were provided by the Holstein Cattle Association of Japan (Tokyo, Japan). The pedigree was untruncated and with 8,654,187 Holsteins born between 1901 and 2023. For animals with missing birth years, the year was estimated as the birth year of their oldest progeny minus three, representing a plausible generation interval for Holsteins [9]. To minimize potential pedigree errors, if parents were recorded as being born in the same year as or later than their progeny, they were set to unknown (n = 57). To estimate F for animals with unknown parents, the unknown parents were assigned to metafounders based on the birth year of their progeny [9, 19], resulting in an additional 123 metafounders being included in the pedigree. Overall, the pedigree included 42,120 sires (including metafounders), of which 64.8% had fewer than ten offspring. In contrast, a small number of sires generated exceptionally large half-sib families, with the maximum reaching 81,618 offspring. In this pedigree, LAP numbers ranged from 0 (for metafounders) to 34 (for young animals), with a mean of 17 and a median of 19.

#### Simulated pedigrees

To evaluate the effects of half-sib family size and pedigree depth on the computational performance of the present algorithm, populations with half-sib family sizes of 1,000, 200, or 4 were simulated using QMSim [21]. The family size of 200 was chosen based on the average of 205.4 observed in the Holstein pedigree. In generation 0 (i.e., base population), 50,000 dams were mated randomly to 100, 500, or 25,000 sires, respectively, producing two litters per dam from different sires (i.e., factorial mating designs). To increase population size to levels comparable to the Holstein pedigree, the numbers of sires and dams were increased at a constant rate of 50% of generation 1 (i.e., 50,000 animals) from generations 2 to 5, resulting in 300,000 animals per generation from generation 5 onward. Each generation, the oldest 50% of sires and 30% of dams were replaced by animals with the highest estimated breeding values from the previous year for a trait with heritability of 0.3. The simulations were continued until 35 generations and replicated three times for each scenario, yielding pedigree files containing 10,000,000 animals in addition to those in generation 0.

### Evaluation of performance

We implemented the proposed modified indirect method (MI), along with the algorithms of Meuwissen and Luo (ML) [12], Sargolzaei and Iwaisaki (SI) [15], and Sargolzaei et al.’s indirect method (I) [13], in Fortran 90. The source code for the proposed method is provided in Additional file 1, Code S1. All algorithms were compiled using the Intel® Fortran Compiler Classic (ifort; version 2021.13.0) with flags -O3 and -Qopenmp. Additionally, the recursive algorithms (REC) proposed by Aguilar and Misztal [9] were executed using the software INBUPGF90.

Computational tests were conducted on a machine equipped with an AMD® Ryzen™ 9 5950X 16-Core 64-bit processor operating at 3.40 GHz, with 8Mb of L2 and 64 Mb of L3 cache. The available physical memory was 64 GB. The wall-clock time for the MI, ML, SI, and I methods on the Holstein pedigree was measured five times using the intrinsic function omp_get_wtime, under 1–32 threaded execution environments. For the simulated pedigrees, computation time was measured for each of the three replicates per scenario, with the program run using 16 threads. The measurements excluded the time required for pedigree sorting, and for preparing the reduced pedigree in the I method. The times were generally short (< 0.5s and < 0.1s, respectively, for the Holstein pedigree) and considered negligible.

For REC, due to the single-threaded implementation in INBUPGF90, only one thread could be used; however, the calculated coefficients were efficiently stored in memory. The results computed from all algorithms were validated to be consistent with each other.

## Results and discussion

Table 1 shows the computation performance of the tested algorithms on the Holstein pedigree. Under single-threaded execution, the MI method reduced computation time to about 7% of that required by the I method (7.24 s vs. 103.40 s; averaged over 3 replications). The improvement is primarily due to (1) a more efficient approach for deriving ${\mathbf{L}^{\prime}_{\mathbf{s},:}}$ in lieu of backward substitution, and (2) recursive functions that reduced the number of elements computed and stored during forward substitution. In this pedigree, the number of computed elements (1.2 × 10^8^ elements) during forward substitution was only 0.2% of that in the I method (6.0 × 10^10^ elements), and this ratio is expected to decline further when population size grows or when animals are more inbred.

**Table 1:** Computation times (seconds ± SD) for the tested algorithms on the Holstein pedigree (n = 8,654,187) under single or multi-threaded execution

Threads	ML^a^	SI^b^	I^c^	MI^d^	REC^e^
1	2294.85 ± 59.40	86.16 ± 4.75	103.40 ± 2.24	7.24 ± 0.20	66.47 ± 0.62
4	604.08 ± 30.78	28.16 ± 1.19	70.53 ± 8.57	2.66 ± 0.16	–
8	308.83 ± 16.15	14.69 ± 0.25	65.50 ± 3.15	1.71 ± 0.13	–
16	159.11 ± 3.41	8.10 ± 0.12	66.34 ± 0.94	1.35 ± 0.06	–
32	89.13 ± 0.36	5.28 ± 0.02	77.50 ± 0.45	1.13 ± 0.01	–

^a^Method proposed by Meuwissen and Luo [12]

^b^Method proposed by Sargolzaei and Iwaisaki [15]

^c^Indirect method proposed by Sargolzaei et al. [13]

^d^Modified indirect method proposed in this study

^e^Recursive algorithm with memorized coefficients proposed by Aguilar and Misztal [9]

With 32 threads, the MI method completed computation in 1.13s on average over 3 replications, approximately 16% of the time required in single-threaded execution (Table 1). Compared to the I method, it exhibited much better scalability under parallel execution. This difference is likely due to the increased risk of cache thrashing in the I method, which computed and stored a large number of elements during each iteration. For example, for sires with LAP numbers of 30, the I method stored an average of 4,260,434 elements in the forward substitution step, corresponding to approximately 32.50Mb of memory when saved as double precision. In contrast, the MI method only stored 5,455 elements (0.04Mb). Since each thread maintained its own copy, the total memory demand for the I method can easily exceed the capacity of the shared CPU cache (64Mb L3 cache in our system).

The MI method also outperformed the ML, SI, and REC methods (Table 1). In fully complete pedigrees without coancestry, the computation times of the SI and ML methods are known to increase exponentially with the number of generations [15]. In contrast, recursive algorithms such as REC or Tier’s algorithm [11] avoid redundant calculations by storing previously computed relationship coefficients. As long as large half-sib families are present in the population, this provides an advantage when the number of generations increases [12]. Consequently, for the tested Holstein pedigree, the REC method performed better than the SI method under single-threaded execution. However, the MI method also temporarily stores the corresponding rows of **L** for sires to minimize redundant calculations, thereby taking advantage of the presence of large half-sib families. Furthermore, because each row of **L** is computed independently, parallelization of the MI method (as well as the I, ML, and SI methods) is more straightforward than that of REC.

**Fig. 1:**
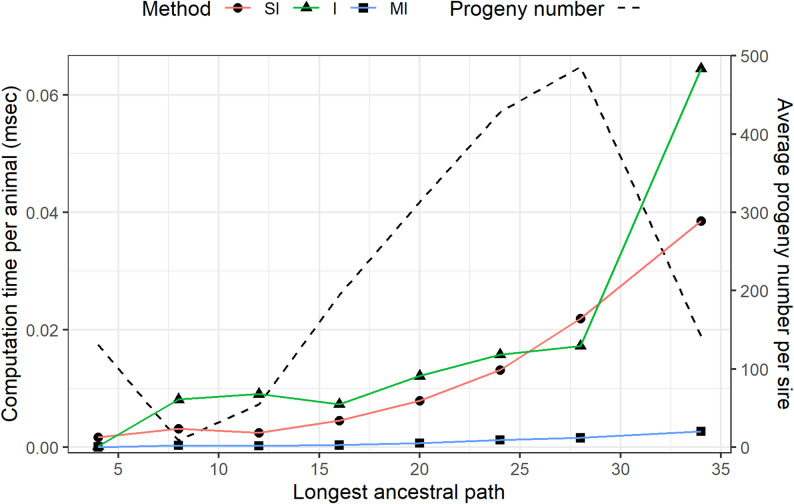
Averaged computation times per animal (in milliseconds) grouped by the longest ancestral path number (solid lines), alongside the average half-sib family sizes (i.e., progeny number per sire) in each group (dotted line) for the Holstein pedigree. SI: Method of Sargolzaei and Iwaisaki [15]. I: Indirect method proposed by Sargolzaei et al. [13]. MI: Modified indirect method proposed in this study

Figure 1 shows the computation time per animal grouped by the LAP numbers for the SI, I, and MI methods (solid lines), alongside the average progeny number of sires (i.e., average half-sib family size) in each group (dotted line). The computation time of the SI method increased exponentially with LAP, suggesting that the performance gap between the SI and MI methods would widen as pedigrees accumulate over generations. Conversely, the I method was particularly inefficient in the last generations, where sires had fewer progenies on average. When a sire has few mates, the ancestral lists of his mates are less likely to overlap, reducing the opportunity to eliminate redundant computations in the I method. Although the MI method is also influenced by family size, its ability to identify and compute only necessary elements allows it to maintain high efficiency even when small families exist.

Table 2 presents simulation results. Across most tested scenarios, the MI method showed superior performance, while the REC method could not complete computations under the available physical memory (64 GB). Compared with the SI method, MI was more resistant to increases in generation number, particularly in populations with large half-sib families. For populations with half-sib family sizes of 1,000 and 200 over 35 generations, the MI method (averaged computation time for 3 simulated pedigrees: 1.91s and 13.43s) required only 5.2% and 4.5% of the time needed by the SI method (36.99s and 301.35s), respectively.

**Table 2:** Computation times (seconds ± SD) for the tested algorithms on simulated pedigrees with half-sib family sizes (i.e., progeny number per sire) of 1,000, 200, and 4. A single thread was used for the recursive algorithm (REC), whereas 16 threads were used for the other algorithms

Family size	Sire number^a^	Generation evaluated	N	SI^b^	I^c^	MI^d^	REC^e^
1000	300	0–15	4,050,100	0.85 ± 0.11	0.55 ± 0.13	0.24 ± 0.03	67.48 ± 2.61
		0–25	7,050,100	8.96 ± 1.68	1.81 ± 0.32	0.93 ± 0.25	196.88 ± 0.57
		0–35	10,050,100	36.99 ± 2.23	4.26 ± 0.53	1.91 ± 0.03	422.95 ± 5.10
200	1,500	0–15	4,050,500	1.54 ± 0.27	2.56 ± 0.33	0.59 ± 0.14	203.78 ± 9.10
		0–25	7,050,500	33.80 ± 2.37	8.67 ± 0.73	3.83 ± 0.19	1000.24 ± 7.06
		0–35	10,050,500	301.35 ± 25.22	21.52 ± 4.00	13.43 ± 1.13	NA^f^
4	75,000	0–15	4,075,000	3.62 ± 0.16	285.67 ± 29.97	2.78 ± 0.47	NA
		0–25	7,075,000	191.35 ± 12.36	983.02 ± 73.57	149.04 ± 7.71	NA
		0–35	10,075,000	4664.37 ± 289.49	2406.80 ± 111.93	2223.95 ± 247.46	NA

^a^Sire number per generation from generation 5 onward

^b^Method proposed by Sargolzaei and Iwaisaki [15]

^c^Indirect method proposed by Sargolzaei et al. [13]

^d^Modified indirect method proposed in this study

^e^Recursive algorithm with memorized coefficients proposed by Aguilar and Misztal [9]

^f^Not available due to physical memory constraints

Compared with the I method, MI exhibited better performance when the pedigree contained many sires but spanned relatively fewer generations. For the population with 75,000 sires per generation (i.e., half-sib family size of 4), the MI method (2.78s) required only 1.0% of the time needed by the I method (285.67s) up to generation 15. However, under this scenario, the advantage of MI diminished rapidly as generations grew. This tendency has not been observed in Fig. 1, where the MI method performed well even for Holsteins with large LAP numbers. This can be attributed to the presence of inbreeding, which reduces the number of genetic origins and, consequently, the number of elements in $\mathbf{y}$ that need to be computed.

Unlike the simulated populations, real livestock pedigrees include animals with variable half-sib family sizes (e.g., small families for young or foreign sires) and pedigree depths (e.g., short due to missing ancestry but long for influential sires), conditions under which the MI method is expected to perform particularly efficiently.

## Conclusions

The proposed algorithm outperforms previous methods by efficiently identifying the necessary elements for computing F and avoiding redundant computations through temporary storage of the computed results. It is well-suited for real livestock populations bred via artificial insemination, where some sires have plentiful progenies, but small families may also exist. The algorithm performs efficiently under parallel execution, making it particularly useful when F need to be computed repeatedly across iterations.

## Supplementary Information

Below is the link to the electronic supplementary material.

Supplementary Material 1

Supplementary Material 2

Supplementary Material 3

## Data Availability

Not applicable.
